# Mass Spectrometric Analysis of Free Methionine Oxidation Levels in *E. coli*

**DOI:** 10.1021/jasms.6c00210

**Published:** 2026-10-05

**Authors:** Maria Ahmed, Philip Bellomio, Bruno Manta, Kyle Swovick, Kevin A. Welle, Jennifer R. Hryhorenko, Sina Ghaemmaghami

**Affiliations:** † Department of Biology, 6927 University of Rochester, Rochester, New York 14627, United States; ‡ 123939 Institut Pasteur Montevideo, Montevideo 11400, Uruguay; § University of Rochester Mass Spectrometry Resource Laboratory, Rochester, New York 14642, United States

**Keywords:** Methionine oxidation, Proteomics, Mass spectrometry (MS)

## Abstract

Oxidation of free methionine plays important roles in cellular redox homeostasis, yet its accurate quantification has been hindered by methodological challenges. Here, we introduce free methionine oxidation by blocking (fMObB), a mass spectrometry-based method that enables accurate measurement of the fractional oxidation of free methionines. Applying fMObB to *E. coli*, we quantify free methionine oxidation under basal and oxidative stress conditions and in strains lacking methionine sulfoxide reductases. We found that during oxidative stress, free methionines exhibit higher oxidation levels than protein-bound methionines and that methionine sulfoxide reductases play a central role in maintaining reduced free methionine pools. Together, this work establishes fMObB as a generalizable strategy for probing free methionine redox states in cellular systems.

## Graphical abstract

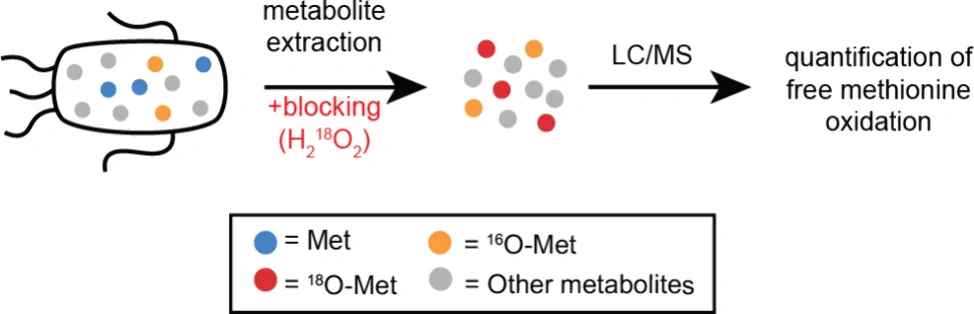

## Introduction

Within cells, methionines (Mets) can be oxidized to methionine sulfoxides (MetOs) by reactions with reactive oxygen species (ROS) or by enzymatic oxidation catalyzed by monooxygenases.^1^ MetOs can subsequently be converted back to Mets by a conserved class of reducing enzymes known as methionine sulfoxide reductases (MSRs).^2,3^ Oxidation of protein-bound Met residues frequently leads to protein misfolding and has been associated with several neurodegenerative diseases and pathogenic aging.^4–6^ Beyond their role in protein damage and repair, cycles of oxidation and reduction of Met residues contribute broadly to cellular redox homeostasis by functioning as an ROS scavenging mechanism. In this context, Met oxidation followed by MSR-mediated reduction consumes NADPH to eliminate ROS, allowing Mets to serve as a sink for reactive oxidants that would otherwise be damaging to other essential cellular components.^7,8^ Moreover, Met oxidation has been shown to act as a regulatory switch for certain oxidative-responsive transcription factors and modulate the activity of a number of enzymes.^9,10^

In comparison to protein-bound Met residues, the oxidation and reduction of free Mets have received less attention. This is despite the fact that free Mets can be as oxidizable as their protein-bound counterparts and that such oxidation can play a critical role in cellular signaling and disease.^11–14^ Although most MSRs, including MsrA and MsrB, preferentially reduce protein-bound MetOs, they can also act on free MetOs with reduced efficiencies.^15^ Additionally, some bacteria encode specialized MSRs dedicated to reducing free MetO.^16,17^ Free MetOs are biologically important metabolites that can function as scavengers similar to their protein-bound counterparts, serve as electron acceptors for the respiratory chain under anaerobic conditions, act as signaling molecules, and provide precursors for methionine biosynthesis.^18^

Despite these important roles, with a few exceptions,^19,20^ the oxidation levels of free Met pools have not been systematically quantified *in vivo*. Although Mets and MetOs are readily detectable by mass spectrometry, accurate quantification of their relative *in vivo* abundances presents several technical challenges that have limited their analyses. First, Mets are highly susceptible to oxidation during sample handling and analysis, making it difficult to distinguish MetOs formed *in vivo* from those generated artifactually during mass spectrometric workflows.^21–23^ Second, differences in ionization efficiencies between Mets and MetOs preclude direct measurement of fractional oxidation from their respective signal intensities alone, necessitating the use of spike-in standards or alternative normalization strategies.^19^

To address these limitations, a method termed methionine oxidation by blocking (MObB) has recently been developed to facilitate the mass spectrometric analysis of Met oxidation levels.^6,23–25^ In this approach, unoxidized Mets are fully oxidized *in vitro* using ^18^O-labeled hydrogen peroxide, and subsequent mass spectrometric measurements of ^18^O- and ^16^O-labeled methionine sulfoxides are used to determine *in vivo* fractional oxidation levels. Although MObB has been successfully applied to quantify Met oxidation within individual proteins and in large-scale proteomic studies,^6,23–27^ it has not been previously used to measure oxidation levels of free Mets.

Here, we describe a modified version of the MObB methodology, termed free methionine oxidation by blocking (fMObB), that enables quantitative measurement of the fractional oxidation of free Mets. As a first application of this approach, we quantified free Met oxidation levels in the cytosol of *E. coli* under basal and oxidative stress conditions. The results provide insights into methionine redox homeostasis in *E. coli* and establish fMObB as a generalizable strategy for assessing free methionine oxidation levels in diverse cellular systems.

## Results and Discussion

### fMObB Can Accurately Quantify Fractional Oxidation of Free Mets

The detailed experimental workflow of fMObB is provided in the Methods section (see Supporting Information). Briefly, cellular extracts containing free Mets and MetOs are treated with ^18^O-labeled hydrogen peroxide, stoichiometrically converting all unoxidized Mets to ^18^O-labeled MetOs. Endogenously formed ^16^O-MetOs and *in vitro*-generated ^18^O-MetOs differ in mass but exhibit identical chromatographic retention times and ionization efficiencies. Consequently, the relative intensities of their mass spectrometric signals provide quantitative measures of fractional Met oxidation within the extract.

To assess the quantitative accuracy of fMObB, we first generated defined mixtures of free ^16^O- and ^18^O-MetOs and experimentally measured their relative abundances (Figure 1A). ^16^O-MetO was obtained commercially, while ^18^O-MetO was prepared in-house by complete oxidation of Mets using ^18^O-labeled hydrogen peroxide. The oxidation and isotopic purity of both stock solutions was confirmed by liquid chromatography-mass spectrometry (LC-MS) (Figure 1B). The two stock solutions were mixed at defined ratios and relative abundances of ^16^O- and ^18^O-MetOs were quantified in triplicate samples (Figure 1C). The experimentally measured ratios showed excellent agreement with expected values (slope = 0.97, R^2^ > 0.99), validating the overall quantitative accuracy of fMObB measurements. However, our experiments detected a background level of ∼2% ^16^O-MetO even in samples expected to contain only ^18^O-MetO. This background likely arises from residual oxidation of the commercially obtained Met used to synthesize ^18^O-MetO, or from isotopic impurity in the ^18^O-labeled hydrogen peroxide. Depending on which source predominates, the fractional oxidation values reported in the following studies may need to be corrected by 0–2% to reflect true *in vivo* levels.

**Figure 1:**
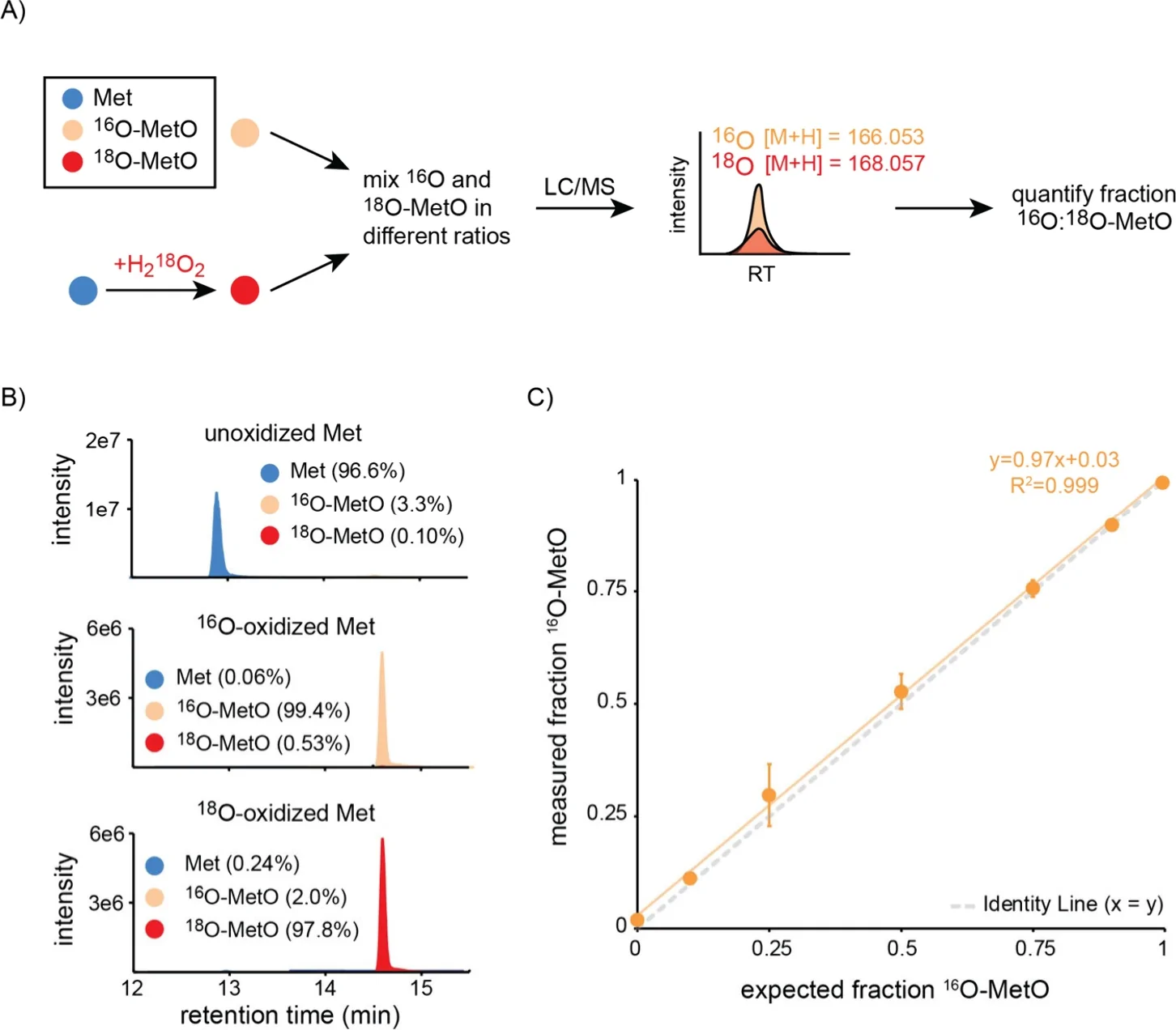
(A) Schematic of the workflow for assessing the accuracy of fMObB. (B) Relative levels of Met, ^16^O-MetO, and ^18^O-MetO in commercially obtained unoxidized Met and ^16^O-MetO, and in-house generated ^18^O-MetO samples. (C) Scatter plot indicating the correlation between measured and expected fraction ^16^O-MetO in defined mixtures of the ^16^O-MetO and ^18^O-MetO.

### Free MetO levels in *E. coli*

We next applied fMObB to measure MetO levels in *E. coli* under varying conditions. Two different strains of *E. coli* were used in the analysis: a wildtype (WT) reference strain (LCB303) and a previously established mutant strain in which all 5 known bacterial MSRs had been deleted (MB6565, here referred to as -MSR).^28,29^ Using a data independent acquisition (DIA) proteomic workflow, we first verified that -MSR cells had depleted levels of MSR expression **(** Figure 2A). Next, WT and -MSR strains were cultured under normal growth conditions or exposed to oxidative stress by the addition of non-lethal concentrations (100 μM) of hypochlorous acid (HOCl) for 1 h. Extracts were obtained from the cells, and fractional oxidation of free Mets was measured by fMObB in triplicate measurements **(** Figure 2 **B–C)**. The efficiency of ^18^O blocking was >99% for all samples.

**Figure 2:**
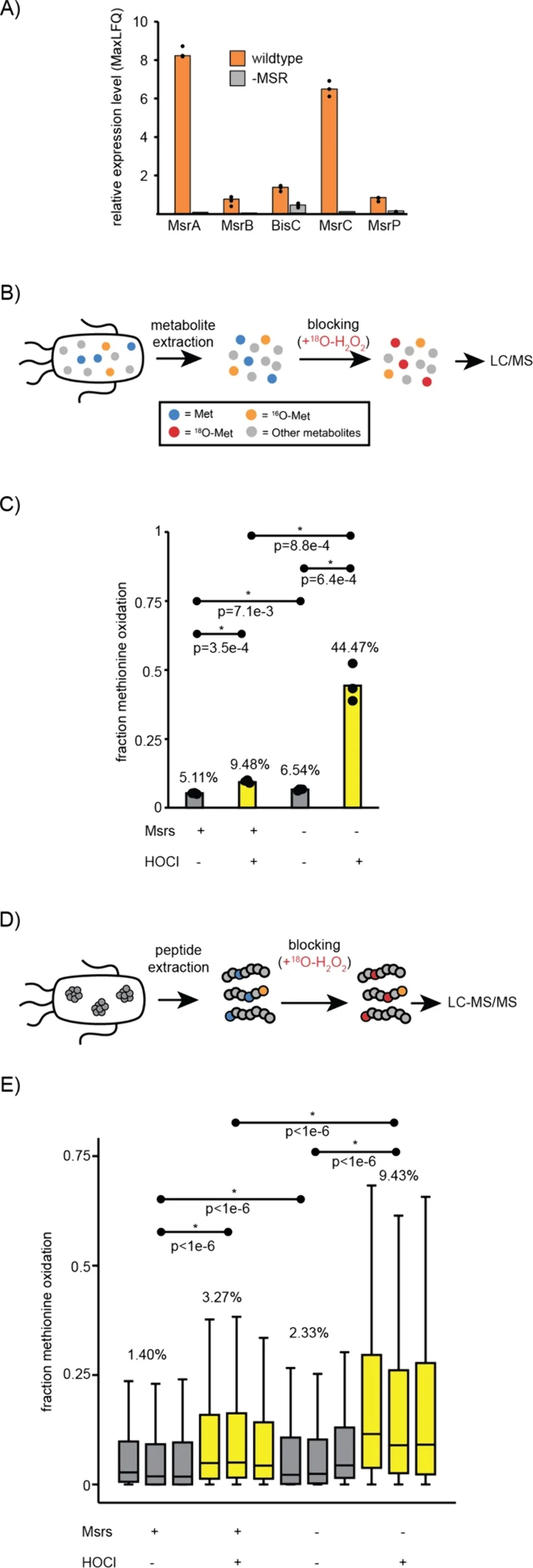
(A) Relative expression levels of MsrA, MsrB, BisC, MsrC and MsrP in unstressed wildtype and -MSR *E. coli* based on MaxLFQ measurements. (B) Schematic of the fMObB methodology for measurement of free methionine oxidation levels. (C) Fractional oxidation levels of free methionines in wildtype and -MSR *E. coli* in the absence and presence of hypochlorous acid (+HOCl). Medians of three replicate measurements are shown. p-values were calculated using two-tailed, equal variance t-test. (D) Schematic of the MObB methodology for measurement of protein-bound methionine oxidation levels. (E) Distributions of fractional oxidation levels of protein-bound methionines in wildtype and -MSR *E. coli* in the absence and presence of hypochlorous acid (+HOCl). The box plots indicate the range, medians, and interquartile range of three replicate experiments. p-values were calculated by applying the Mann–Whitney U test to the average of replicate experiments across the four conditions. The indicated percentages are the average percentage of methionines that are oxidized in each condition.

Under basal growth conditions, WT cells exhibited a free methionine oxidation level of 5.1% (±0.2%). In contrast, cells lacking MSRs displayed a significantly higher basal MetO level of 6.5% (±0.4%) (two-tailed, equal-variance t-test, *p* = 0.007). Exposure to HOCl resulted in a marked increase in methionine oxidation in both strains, with free MetO levels rising to 9.6%(±0.6%) in WT cells and 45% (±7%) in the MSR-deficient strain. Together, these data establish a basal free methionine oxidation level of 3% to 5% in *E. coli* (taking into account the background oxidation described above) and demonstrate that oxidative stress dramatically elevates free methionine oxidation. Moreover, the pronounced increase observed in the absence of MSRs indicates that, collectively, MSRs play a substantial role in maintaining reduced free methionine pools, particularly under conditions of oxidative stress.

### Protein-Bound MetO Levels in *E. coli*

We next evaluated how the oxidation levels of free Mets in *E. coli* compare to protein-bound Mets **(** Figure 2D-E). Protein extracts from the experiments described above were analyzed using the previously described MObB protocol^6^ to globally measure the oxidation of Mets throughout the proteome. The efficiency of ^18^O blocking was >98% for all samples. Between three replicate experiments, we were able to measure the oxidation levels of ∼8,000 methionine residues mapped to ∼2,400 proteins. Distributions of measured oxidation levels are plotted in Figure 2E. Similar to the fMObB analyses above, we observed that oxidative stress and deletion of MSRs significantly increase the fractional oxidation of protein-bound Met residues. However, these increases were significantly less pronounced than free Mets. Under oxidative stress conditions in the -MSR strain, the average oxidation level of Met residues in the proteome was 9.4%.

Direct quantifications of free MetO are scarce and have been largely confined to human plasma, where fractional oxidation has been reported to be approximately 10%.^19,20^ The basal levels we measure in the *E. coli* cytosol are considerably lower. This difference may reflect both the oxidizing character of the extracellular environment relative to that of the bacterial cytosol and the susceptibility of HPLC and GC-MS workflows to *ex vivo* oxidation. To our knowledge, no previous measurement of free MetO has employed a blocking strategy to control for this artifact.

Our protein-bound MetO measurements can be compared more directly with prior work employing the ^18^O blocking chemistry. Median protein-bound oxidation in unstressed human fibroblasts was 3.5–5.0%, with comparably low levels in mouse brain, in close agreement with our basal values.^6,23^ Applying an analogous blocking approach to the secretome of the white-rot fungus *Pycnoporus cinnabarinus*, a mean oxidation of ∼18% was measured.^26^ As MSRs are not secreted, the fungal secretome constitutes a natural analog of our -MSR strain and supports the central role of MSRs in maintaining reduced protein-bound methionines. Additionally, bulk analyses of unstressed plant leaves report 2–6% MetO rising to over 50% under stress.^30–32^

## Conclusion

Our results indicate that oxidative stress and loss of MSR activity lead to substantially higher oxidation levels of free Met than of protein-bound Met. These observations raise several important questions that warrant further investigations. First, it remains unclear whether the elevated oxidation of free Mets relative to protein-bound Mets reflects possible differences in intrinsic susceptibility to oxidation, a stronger dependence on MSR-mediated reduction, or the formation from degradation of oxidized proteins. Second, the relative contributions of individual MSRs to the reduction of free versus protein-bound methionine sulfoxides under oxidative-stress conditions remain unresolved. It is known that some MSRs exhibit higher *in vitro* catalytic efficiencies toward protein-bound methionine sulfoxides, whereas others preferentially reduce the level of free methionine sulfoxides. For example, MsrC acts exclusively on free Met-R-O and shows among the highest *in vitro* activities recorded for any MSR against this substrate.^16^ However, how these specificities translate to net cellular activity is not well understood. As highlighted by our proteomic analyses (Figure 2A), different MSRs are expressed at markedly different levels in *E. coli*, suggesting that their relative cellular abundances may strongly influence their overall contributions to methionine redox homeostasis. Future studies applying fMObB and MObB to strains lacking individual MSRs or defined combinations thereof will help clarify the division of labor within this enzyme family. More broadly, the fMObB approach established here provides a generalizable framework for probing the redox state of free methionine pools across diverse biological systems.

## Supplementary Material



## Notes

Experimental methods including standard preparation, bacterial culture and treatment, metabolite extraction, proteomic sample preparation, mass spectrometry parameters, and data analysis (PDF)

## Abbreviations

Met: methionine
MetO: methionine sulfoxide
ROS: reactive oxygen species
MSR: methionine sulfoxide reductase
MsrA: methionine sulfoxide reductase A
MsrB: methionine sulfoxide reductase B
MObB: Methionine Oxidation by Blocking
fMObB: free Methionine Oxidation by Blocking
DIA: data independent acquisition
HOCl: hypochlorous acid
BEH: bridged ethyl hybrid
H-ESI: heated electrospray ionization
AGC: automatic gain control
TEAB: triethylammonium bicarbonate
FWHM: full-width at half maximum
RF: radio frequency
BCA: bicinchoninic acid
DTT: dithiothreitol
IAA: iodoacetamide
TFA: trifluoroacetic acid
ACN: acetonitrile
AH: ammonium hydroxide
HCD: higher-energy collisional dissociation
DDA: data dependent acquisition
MIPS: Monoisotopic Precursor Selection
MBR: match-between-runs
MaxLFQ: maximal label-free quantification
