# Supplementary material for: Mass Spectrometric Analysis of Free Methionine Oxidation Levels in E. coli
Source: J Am Soc Mass Spectrom. 2026 Oct 5;37(11):2669–73. doi: 10.1021/jasms.6c00210 (PMC13741921; doi:10.1021/jasms.6c00210)
Supplement: Supplementary file 1 [file js6c00210_si_001.pdf]

Supporting Information

Mass spectrometric analysis of free methionine oxidation levels in *E. coli*

Maria Ahmed[1], Philip Bellomio[1], Bruno Manta[2], Kyle Swovick[3], Kevin A. Welle[3], Jennifer R. Hryhorenko[3], Sina Ghaemmaghami[1,3*]

[1]Department of Biology, University of Rochester, Rochester, NY, 14627, USA

[2] Institut Pasteur Montevideo, 11400, Montevideo, Uruguay

[3]University of Rochester Mass Spectrometry Resource Laboratory, Rochester, NY, 14642, USA

[*]Correspondence:

Sina Ghaemmaghami
sina.ghaemmaghami@rochester.edu
585-275-4829,
326 Hutchison Hall
University of Rochester
Rochester NY 14627

Methods

Preparation of ^{16}O and ^{18}O-MetO standards

^{16}O-MetO stocks were prepared by dissolving L-MetO (Sigma) in water to a final concentration of 100 mM. To generate ^{18}O-MetO, L-methionine (Fisher) was dissolved in water to a concentration of 200 mM and oxidized with 0.25 M $H_2{}^{18}O_2$ (Sigma) for 30 minutes at 37°C, then frozen and lyophilized. The resulting ^{18}O-MetO was brought up to a final concentration of 100 mM in water. Before mixing these two MetO species to generate the standard curve, ^{16}O- and ^{18}O-MetO stocks were diluted to 10 mM working concentrations. These working stocks were then mixed in different ratios (0:100, 10:90, 25:75, 50:50, 75:25, 90:10, 100:0) of ^{16}O:^{18}O-MetO in a final 20 μL volume.

Bacteria culture and treatment

An *E. coli* strain derivative from MC4100 auxotroph for methionine (ΔmetB) lacking the genes for the five known prokaryotic MSRs – MsrA, MsrB, MsrC, BisC, and MsrP – named MB6565,[1] and its reference strain MC4100 ΔmetB (named LCB303[2]) were streaked onto LB agar plates and incubated overnight at 37°C. Single colonies from these plates were used to inoculate a 5 mL culture in LB media, which were grown overnight at 30°C, shaking at 150 rpm due to the sensitivity of MB6565 to oxidative stress. The OD_{600} was taken of these overnight cultures before pelleting at 4000 xg for 5 minutes. Cells were washed with 1 mL 1x PBS (Corning) and spun down again at 4000 xg for 5 minutes. Cells were resuspended in 1x PBS to get an inoculum with an OD_{600} of 1. This resuspension was diluted 10x in 5 mL of minimal M9 media with 200 μM Met, inoculating two sets of three replicates for each strain. After 23 hours of growth, either 50 μL of 10 mM sodium hypochlorite (Sigma) or 50 μL 1X PBS was added and briefly vortexed prior to the final hour of incubation. The OD_{600} of the cultures was measured after this treatment period, and cultures were pelleted by centrifugation at 4000 xg for 5 minutes. Cells were washed twice with 1x PBS, pelleted again, and resuspended in PBS to obtain an OD_{600} of 1. For metabolomic analysis, 1 mL of the cell suspension was transferred to a new microcentrifuge tube, pelleted, and frozen in liquid nitrogen before storing at -80°C. The rest of the resuspension was pelleted in a separate microcentrifuge tube and frozen at -80°C before proteomic analysis.

Metabolite extraction and blocking

Pellets for metabolomics were resuspended in 1 mL of 80:20 methanol to water metabolite extraction solution that was chilled on dry ice. Cells were kept on ice and lysed by sonication at 50% power, 15 seconds on and 30 seconds off for 3 cycles. The sample was transferred to dry ice for 15 minutes, then placed on regular ice for 30 minutes with a 5-second vortex every 10 minutes. Metabolite extract was separated from other cellular materials through a 10-minute centrifugation at 19,000 xg at 4°C. 800 µL of the supernatant was transferred to a new microcentrifuge tube and lyophilized. Metabolite extracts were resuspended in 25 µL of 0.1% trifluoroacetic acid (TFA) water. 25 µL of 1% $H_2{}^{18}O_2$ solution, prepared by diluting 3% $H_2{}^{18}O_2$ in 0.1% TFA water, was added to reach a final concentration of 0.5% $H_2{}^{18}O_2$ (v/v) in each sample. Samples were oxidized by incubating at 37°C for 30 minutes with $H_2{}^{18}O_2$, then frozen at -80°C and lyophilized. Samples were reconstituted in 50% acetonitrile (A955, Fisher Scientific) and transferred to glass vials for LC/MS analysis.

Mass spectrometry of metabolite extracts

For LC-MS analysis, metabolite extracts were analyzed by high-resolution mass spectrometry with an Orbitrap Exploris 240 (Thermo) coupled to a Vanquish Flex liquid chromatography system (Thermo). Samples were injected on a Waters XBridge XP BEH Amide column (150 mm length × 2.1 mm id, 2.5 µm particle size) maintained at 25°C, with a Waters XBridge XP VanGuard BEH Amide (5 mm × 2.1 mm id, 2.5 µm particle size) guard column. Mobile phase A was 100% LC-MS grade H2O with 10 mM ammonium formate and 0.125% formic acid. Mobile phase B was 90% acetonitrile with 10 mM ammonium formate and 0.125% formic acid. The gradient was 0 minutes, 100% B; 2 minutes, 100% B; 3 minutes, 90% B; 5 minutes, 90% B; 6 minutes, 85% B; 7 minutes, 85% B; 8 minutes, 75% B; 9 minutes, 75% B; 10 minutes, 55% B; 12 minutes, 55% B; 13 minutes, 35%, 20 minutes, 35% B; 20.1 minutes, 35% B; 20.6 minutes, 100% B; 22.2 minutes, 100% B all at a flow rate of 150 µl min^{-1}, followed by 22.7 minutes, 100% B; 27.9 minutes, 100% B at a flow rate of 300 µl min_{-1}, and finally 28 minutes, 100% B at flow rate of 150 µl min^{-1}, for a total length of 28 minutes. The H-ESI source was operated in positive mode at spray voltage 3500 with the following parameters: sheath gas 35 au,

aux gas 7 au, sweep gas 0 au, ion transfer tube temperature 320°C, vaporizer temperature 275° C, full scan MS1 mass resolution of 120,000 FWHM, RF lens at 70%, and standard automatic gain control (AGC).

Proteomic sample preparation and blocking

Pellets collected for proteomic analysis were lysed with 50 µL of lysis buffer (5% SDS in 50 mM triethylammonium bicarbonate (TEAB) by sonication. Sonication was performed at 4°C in 30-second intervals at 50% power three times with 30-second breaks on ice in between. Samples were spun at 19,000 xg for 15 minutes at 4°C, and the supernatant was saved. Protein concentrations were determined by bicinchoninic acid (BCA) assay (Thermo). 25 µg of each sample was transferred to a new 0.5 mL tube, and 5% SDS in 50 mM TEAB was added to reach a total 25 µL volume. Samples were first incubated at 55°C for 60 min with 2.2 µL of 25 mM dithiothreitol (DTT). Samples were then incubated with 2.4 µL of 125 mM iodoacetamide (IAA) in the dark at room temperature for 20 minutes. Subsequently, 3 µL of 12% phosphoric acid was added to each sample along with 195.6 µL of 90% methanol in 100 mM TEAB. Samples were then added 165 µl at a time to S-Trap™ columns, spinning down the sample at 4,000 xg for 1 min in between each addition, and between each subsequent step. Columns were washed twice with 150 µL 90% methanol in 100 mM TEAB twice. 20 µL of 50 ng/uL trypsin was added to each column and allowed to soak for 5 minutes at room temperature before another 20 µL of 50 mM TEAB was added to the column. Samples were incubated at 37°C overnight in a water bath.

After incubation, peptides were eluted first with 40 µL of 0.1% trifluoroacetic acid (TFA) in water and then with 50/50 acetonitrile (ACN): 0.1% TFA water. Samples were frozen at -80°C and then lyophilized. Dried peptides were resuspended in 25 µL of 0.1% trifluoroacetic acid (TFA) water. 25 µL of 1% $H_2{}^{18}O_2$ solution, prepared by diluting 3% $H_2{}^{18}O_2$ in 0.1% TFA water, was added for a final concentration of 0.5% $H_2{}^{18}O_2$ (v/v) in each sample. Samples were oxidized by incubating at 37°C for 30 minutes, then frozen at -80°C and lyophilized.

Fractionation of proteomic samples

For MObB samples, dried down peptides were resuspended in 50 µL of 100 mM ammonium hydroxide (AH). Elution buffers for fractionation were made following the protocol below (Table S1). C18 stage tips were first activated by washing with acetonitrile (ACN) twice and then washing twice with 10 mM AH. Samples were loaded onto the stage tip and then washed once more with 10 mM AH. Fractions 1-8 were eluted with the elution buffer of the corresponding elution number. Every i and i+8 fractions (i.e. fraction 1 and fraction 9) were combined. Each wash and elution step was performed with 50 uL volumes by spinning at 2000 xg for 2 minutes. Fractions were frozen at -80°C and lyophilized. Fractionated samples were brought up in 0.1% TFA in water to 0.25 µg/µl and added to a 96-well sample plate.

Table S1

Details of elution buffers used for fractionation.

Elution Buffer	ACN (%)	ACN (uL)	10 mM AH (uL)
E1	2	20	980
E2	3.5	35	965
E3	5	50	950
E4	6.5	65	935
E5	8	80	920
E6	9.5	95	905
E7	11	110	890
E8	12.5	124	875
E8	14	140	860
E10	15.5	155	845
E11	17	170	830
E12	18.5	185	815
E13	20	200	800
E14	21.5	215	785
E15	27	270	730
E16	50	500	500

Mass spectrometry of proteomic samples

Peptides were injected onto a 75 µm x 2 cm trap column (Thermo Fisher) prior to re-focusing on an Aurora Elite 75 µm x 15 cm C18 column (IonOpticks) using a Vanquish Neo UHPLC (Thermo Fisher), connected to an Orbitrap Astral mass spectrometer (Thermo Fisher). Solvent A was 0.1% formic acid in water, while solvent B was 0.1% formic acid in 80% acetonitrile. Ions were introduced to the mass spectrometer using an Easy-Spray source operating at 2 kV.

For samples analyzed using data-independent acquisition (DIA), the gradient began at 1% B and ramped to 5% B in 0.1 minutes, increased to 30% B in 12.1 minutes, increased to 40% in 0.7 minutes, and finally increased to 99% B in 0.1 minutes and was held for 2 minutes to wash the column for a total runtime of 15 minutes. After each run was completed, the column was re-equilibrated with 1% B prior to the next injection. The mass spectrometer was operated in DIA mode, with MS1 scans acquired in the Orbitrap at a resolution of 240,000, with a maximum injection time of 5 ms over a range of 380-980 *m/z*. DIA MS2 scans were acquired in the Astral mass analyzer with a 3 ms maximum injection time using a variable windowing scheme, using 2 Da windows from 380-680 *m/z*, 4 Da windows from 680-800 *m/z*, and 8 Da windows from 800-980 *m/z*. The HCD collision energy was set to 25%, and the normalized AGC was set to 500%. Fragment ions were collected over a scan range of 150-2000 *m/z*. The cycle time was 0.6 seconds.

For samples analyzed using data-dependent acquisition (DDA), the gradient began at 1% B and ramped to 5% B in 0.1 minutes, increased to 30% B in 21.3 minutes, increased to 40% in 1.5 minutes, and finally increased to 99% B in 0.1 minutes and was held for 2 minutes to wash the column for a total runtime of 25 minutes. After each run was completed, the column was re-equilibrated with 1% B prior to the next injection. The mass spectrometer was operated in DDA mode, with MS1 scans acquired in the Orbitrap and MS2 scans acquired in the Astral. The cycle time was set to 0.5 seconds. Monoisotopic Precursor Selection (MIPS) was set to “Peptide”. The full scan was done over a range of 375-1400 *m/z* with a resolution of 120,000 at *m/z* of 200, a normalized

AGC target of 300%, and a maximum injection time of 10 ms. Peptides with a charge state between 2-5 were picked for fragmentation. Precursor ions were fragmented by high-energy collisional dissociation (HCD) using a collision energy of 28% with an isolation width of 1.1 *m/z*. The fragment scans were done over a range of 125-2000 *m/z* with a normalized AGC target of 300% and a maximum injection time of 10 ms. Dynamic exclusion was set to exclude after 1 time with a 6 second duration and a high and low mass tolerance of 7 ppm. The minimum filter intensity threshold was set to 5,000.

Data analysis

For fMObB samples, LC-MS data were analyzed by Maven software for peak area determination, and metabolites were identified by matching to within-run standards. The spectra for each of the three metabolites of interest (Met, ^{16}O-MetO, ^{18}O-MetO) were specifically isolated for peak area determination by searching for their *m/z* in the protonated state (150.058, 166.053, and 168.057, respectively). The fraction oxidation in fMObB samples was determined by dividing the calculated intensity of ^{16}O-MetO by the sum of the ^{16}O-MetO and ^{18}O-MetO intensity and averaging across three replicates.

For expression analysis of DIA data, the raw data were processed with DIA-NN version 1.9.2 (https://github.com/vdemichev/DIA-NN).[3] For all experiments, data analysis was carried out using library-free analysis mode in DIA-NN. To annotate the library, the *E. coli* strain K-12 UniProt ‘one protein sequence per gene’ database (UP000000625, downloaded 02/10/2025) was used, with ‘deep learning-based spectra and RT prediction’ enabled. For precursor ion generation, the maximum number of missed cleavages was set to 1, maximum number of variable modifications to 1 for Ox(M), peptide length range to 7-30, precursor charge range to 2-3, precursor *m/z* range to 380-980, and fragment *m/z* range to 150-2000. The quantification was set to ‘Robust LC (high precision)’ mode with normalization set to RT-dependent, MBR enabled, protein inferences set to ‘Genes’, and ‘Heuristic protein inference’ turned off. MS1 and MS2 mass tolerances, along with the scan window size were automatically set by the software. Precursors were subsequently filtered at library precursor q-value (1%), library protein group q-value (1%), and posterior error probability (50%). Protein quantification

was carried out using the MaxLFQ intensities obtained from the DIA-NN output files which were averaged across three replicates of the WT strain.

Measurement of protein-bound methionine oxidation levels by MObB were conducted using a DDA workflow as described above. Raw data were searched using MSFragger v 4.1 in FragPipe v. 22.0.[4] The search in FragPipe used a Uniprot *E. coli* database (UP000000625_8333, downloaded 05/05/2025) supplemented with decoys and contaminants by FragPipe. Methionine oxidation with ^{16}O and ^{18}O were set as variable modifications along with N-terminal acetylation, and carbamidomethylated cysteine was set as a fixed modification. A maximum of 3 modifications was allowed per peptide. The precursor mass tolerance was set at 20 ppm. Mass calibration and parameter optimization were enabled along with validation tools. Trypsin was set as the protease with up to 2 missed cleavages allowed. MS1 files were generated from raw files using MSConvert.[5] MS1 files and Fragpipe-generated .psm files were used to measure fractional oxidation levels as previously described.[6, 7] All raw data and search results are available in the ProteomeXchange Consortium via the PRIDE partner repository (accession number PXD076670).[8]

References

(1) Anton, B. P.; Morgan, R. D.; Ezraty, B.; Manta, B.; Barras, F.; Berkmen, M. Complete Genome Sequence of Escherichia coli BE104, an MC4100 Derivative Lacking the Methionine Reductive Pathway. *Microbiol Resour Announc* **2019**, *8* (28). DOI: 10.1128/MRA.00721-19.

(2) Gennaris, A.; Ezraty, B.; Henry, C.; Agrebi, R.; Vergnes, A.; Oheix, E.; Bos, J.; Leverrier, P.; Espinosa, L.; Szewczyk, J.; et al. Repairing oxidized proteins in the bacterial envelope using respiratory chain electrons. *Nature* **2015**, *528* (7582), 409-412. DOI: 10.1038/nature15764.

(3) Demichev, V.; Messner, C. B.; Vernardis, S. I.; Lilley, K. S.; Ralser, M. DIA-NN: neural networks and interference correction enable deep proteome coverage in high throughput. *Nat Methods* **2020**, *17* (1), 41-44. DOI: 10.1038/s41592-019-0638-x.

(4) Kong, A. T.; Leprevost, F. V.; Avtonomov, D. M.; Mellacheruvu, D.; Nesvizhskii, A. I. MSFragger: ultrafast and comprehensive peptide identification in mass spectrometry-based proteomics. *Nat Methods* **2017**, *14* (5), 513-520. DOI: 10.1038/nmeth.4256.

(5) Chambers, M. C.; Maclean, B.; Burke, R.; Amodei, D.; Ruderman, D. L.; Neumann, S.; Gatto, L.; Fischer, B.; Pratt, B.; Egertson, J.; et al. A cross-platform toolkit for mass spectrometry and proteomics. *Nat Biotechnol* **2012**, *30* (10), 918-920. DOI: 10.1038/nbt.2377.

(6) Bettinger, J. Q.; Simon, M.; Korotkov, A.; Welle, K. A.; Hryhorenko, J. R.; Seluanov, A.; Gorbunova, V.; Ghaemmaghami, S. Accurate Proteomewide Measurement of Methionine Oxidation in Aging Mouse Brains. *J Proteome Res* **2022**, *21* (6), 1495-1509. DOI: 10.1021/acs.jproteome.2c00127.

(7) Bettinger, J. Q.; Welle, K. A.; Hryhorenko, J. R.; Ghaemmaghami, S. Quantitative Analysis of in Vivo Methionine Oxidation of the Human Proteome. *J Proteome Res* **2020**, *19* (2), 624-633. DOI: 10.1021/acs.jproteome.9b00505.

(8) Perez-Riverol, Y.; Bandla, C.; Kundu, D. J.; Kamatchinathan, S.; Bai, J.; Hewapathirana, S.; John, N. S.; Prakash, A.; Walzer, M.; Wang, S.; et al. The PRIDE database at 20 years: 2025 update. *Nucleic Acids Res* **2025**, *53* (D1), D543-D553. DOI: 10.1093/nar/gkae1011.
